# Serum prolactin as a biomarker for the study of intracerebral dopamine effect in adult patients with phenylketonuria: a cross-sectional monocentric study

**DOI:** 10.1186/s40001-016-0212-2

**Published:** 2016-05-11

**Authors:** Eszter Juhász, Erika Kiss, Erika Simonova, Attila Patócs, Peter Reismann

**Affiliations:** 2nd Department of Medicine, Semmelweis University, Szentkirályi Street 46, Budapest, 1088 Hungary; 1st Department of Pediatrics, Semmelweis University, Bókay J. Street 53, Budapest, 1083 Hungary; Hungarian Academy of Sciences and Semmelweis University “Lendület” Hereditary Endocrine Tumors Research Group, Szentkirályi Street 46, Budapest, 1088 Hungary

**Keywords:** Phenylketonuria, Prolactin, PKU, Dopamine

## Abstract

**Background:**

It has been previously postulated that high phenylalanine (Phe) might disturb intracerebral dopamine production, which is the main regulator of prolactin secretion in the pituitary gland. Previously, various associations between Phe and hyperprolactinemia were revealed in studies performed in phenylketonuria (PKU) children and adolescents. The aim of the present study was to clarify whether any relation between serum phenylalanine and prolactin levels can be found in adult PKU patients.

**Patients and methods:**

We conducted a cross-sectional, monocentric study including 158 adult patients (male *n* = 68, female *n* = 90) with PKU. All patients were diagnosed during newborn screening and were treated since birth. Serum Phe, tyrosine (Tyr), prolactin (PRL), and thyroid-stimulating hormone (TSH) levels were measured, and Phe/Tyr ratio was calculated. Males and females were analyzed separately because the serum prolactin level is gender-dependent.

**Results:**

No significant correlations were found between serum phenylalanine, tyrosine, or the Phe/Tyr ratio and serum prolactin level either in the male or in the female group.

**Conclusions:**

In treated adult PKU patients, the serum prolactin level may not be significantly influenced by Phe or Tyr serum levels.

## Background

Phenylketonuria (PKU, OMIM: 261600) is the most common amino acid metabolism disorder. In PKU, the deficiency of the hepatic enzyme phenylalanine hydroxylase (PAH) causes elevated serum phenylalanine (Phe) with normal to low tyrosine (Tyr) levels. Untreated PKU leads to mental retardation, psychiatric, and various neurological disorders beyond other complications in affected patients. To achieve the best outcomes, PKU patients need a Phe-low diet and lifelong Phe-free amino acid substitution. In some cases, sapropterin therapy can ease the diet limitation [1].

High phenylalanine impairs intracerebral neurotransmitter availability in multiple ways [2, 3]. On the one hand, Phe can competitively inhibit rate-limiting enzymes in the synthesis of various transmitters (e.g., tyrosine-3 hydroxylase and tryptophan-5-hydroxylase). On the other hand, Phe blocks the transport of transmitter precursors at the neutral amino acid transporter of the blood–brain barrier (BBB) [4–6]. A recent study with rodent PKU model confirmed these pathophysiological disturbances [7]. A study by de Groot et al. showed that increased blood Phe concentrations are associated with reduced blood-to-brain Tyr transport and decreased intracerebral Tyr availability for neurotransmitter synthesis [8].

Obtaining information about the intracerebral neurotransmitter state in PKU is relevant, since the results can influence the treatment strategy. However, direct examination of intracerebral transmitter concentration or availability is not feasible in daily practice. For research purposes, measuring dopamine or its metabolites from intracerebral fluid is possible, but only in selective cases and not as a routine [9]. Therefore, surrogate markers are highly needed that can make a diagnostic impact of the intracerebral neurotransmitter effect in PKU.

Prolactin (luteotropic hormone, PRL), a protein hormone, is synthesized in the anterior pituitary, and its main action is the initiation and maintenance of lactation. Pituitary prolactin secretion is decisively regulated through the tonic inhibitory effect of dopamine secreted from the hypothalamic tuberoinfundibular neurons in the arcuate and periventricular nuclei (A12, 14) [10]. Intracerebral dopamine deficiency results in hyperprolactinemia [11].

In PKU, hyperphenylalaninemia can influence the intracerebral transformation of tyrosine to l-dihydroxyphenylalanine (l-DOPA), which is subsequently converted to dopamine [12]. Previous investigations presumed that elevated Phe levels can increase prolactin secretion through reduced intracerebral dopamine availability [13–15].

Clinical studies performed among children or adolescents have investigated the association between Phe and prolactin in PKU, but the results were conflicting [6, 12, 16].

Therefore, our aim was to assess whether an association between Phe and PRL exists in adult PKU patients.

## Patients and methods

### Patients

In a monocentric, cross-sectional study, 158 adult patients with PKU were consecutively enrolled between January 2014 and March 2015 at the Semmelweis University, Budapest, Hungary. All patients were diagnosed during the neonatal screening program, and their treatment was initiated from birth. Patients were regularly examined at the 2nd Department of Medicine, Semmelweis University, Budapest, Hungary.

Patients with conditions affecting prolactin secretion (pregnancy, breast feeding, thyroid or pituitary disorders, use of contraceptive pills, or drugs known to influence prolactin secretion) were excluded. All procedures followed were in accordance with the ethical standards of the responsible committee on human experimentation (Semmelweis University) and with the Helsinki Declaration of 1975, and the study was approved by the Hungarian ethical committee (ETT TUKEB (Medical Research Council Scientific and Research Committee): reference number: 5075-2/2014/EKU). Informed consent was obtained from all patients for being included in the study.

From the 158 PKU adults, 90 patients were female and 68 were male. The mean age was 30.4 ± 6.1 years. According to the Hungarian PKU Guidelines, the recommended upper target Phe concentration for adulthood is 600 μmol/l. None of the patients were treated with BH4.

### Methods

All blood samples were drawn from the antecubital vein under standardized conditions in fasting state between 08:00 and 10:00 a.m. Phenylalanine and tyrosine levels were measured by API2000 LC/MS/MS at the 1st Department of Pediatrics. The serum concentration of prolactin was determined at the Central Laboratory of Semmelweis University using a chemi-immunometric assay (CMIA, Abbott Architect, Abbott Park, USA). TSH was also checked in order to rule out the TRH-induced hyperprolactinemia using this chemi-immunometric assay (CMIA, Abbott Architect, Abbott Park, USA). In a few elevated prolactin samples, PEG precipitation was used to detect macroprolactin. The reference values for prolactin were 1.4–24 ng/ml in female, 1.6–10.7 ng/ml in males; for TSH was 0.35–4.9 mU/l.

### Statistics

For association studies, Spearman’s rho correlation coefficients was used. For analyzing the differences between different subgroups, Mann–Whitney non-parametric tests were used. Since data were not normally distributed, results are reported as median (minimum–maximum) values. All statistical analysis were performed using SPSS version 23 (IBM Corp. in Armonk, NY, USA).

## Results

The median levels were as follows: Phe: 619 (124–1259) μmol/l; Tyr: 54 (21–169) μmol/l. The Phe/Tyr ratio was 11 (1.6–43.7). The prolactin level was 12 (3–75) ng/ml in the whole studied population. All study participants had normal TSH results.

Since the serum prolactin level is gender-dependent, our patient group was divided into male and female groups and the associations were studied separately. Patient characteristics are included in Table 1. The median age of the female group was 32 (18–49) years, while in the male group, it was 31 (19–44) years. The median concentrations of Phe, Tyr, and PRL were as follows: Female group, Phe: 574 (124–1221) μmol/l, Tyr: 45 (21–169) μmol/l; PRL: 14 (5–75) ng/ml; Male group, Phe: 642 (253–1259) μmol/l, Tyr: 55 (24–127) μmol/l; PRL: 9 (4–47) ng/ml. None of the patients had prolactin-related complaints or symptoms, such as menstrual irregularity, galactorrhea, or erectile dysfunction.

**Table 1:** Median age and serum concentration of Phe, Tyr, Phe/Tyr, TSH, and PRL in adult patients with PKU

	Female	Male
Number of patients	90	68
Age [years] (min–max)	32 (18–49)	31 (19–44)
Phe [μmol/l] (min–max)	574 (124–1221)	642 (253–1259)
Tyr [μmol/l] (min–max)	45 (21–169)	55 (24–127)
Phe/Tyr	11.9 (1.6–43.7)	11 (2.4–39.5)
Prolactin [ng/ml] (min–max)	14 (5–75)	9 (4–47)
TSH [mU/l] (min–max)	1.5 (0.6–4.3)	1.1 (0.4–4.9)

TSH normal range 0.35–4.9 mU/l; Prolactin: female: 1.4–24 ng/ml; male: 1.6–10.7 ng/ml

It has to be mentioned that in the female group the median Phe concentration was slightly below, whereas in the male group the median Phe level was slightly above the recommended upper target Phe concentration for adulthood (600 μmol/l). Comparing the gender groups, males had significantly higher Phe levels (*p* = 0.018), but the Tyr level (*p* = 0.120) and the Phe/Tyr ratio (*p* = 0.274) did not differ significantly between groups.

The highest prolactin value was 75 ng/ml, but this level alone without any clinical complaint does not represent a requirement for further endocrinological investigations. In samples showing a higher PRL concentration, PEG precipitation was used to detect macroprolactin, but none of these samples were found to be positive.

**Fig. 1:**
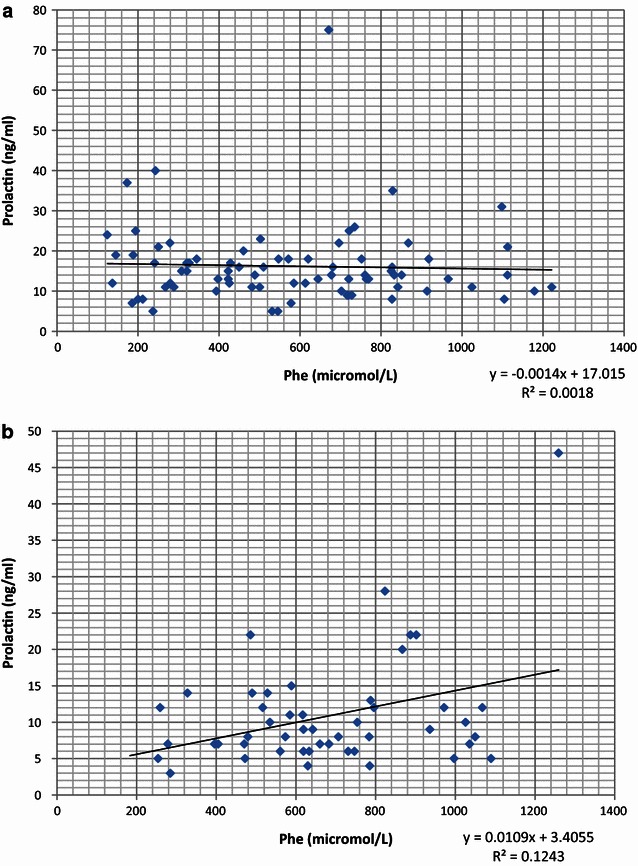
**a** Graphical presentation of the Phe-PRL relationship in the female PKU adult subgroup. **b** Graphical presentation of the Phe-PRL relationship in the male PKU adult subgroup

In the male group, neither phenylalanine nor tyrosine serum concentration, nor the Phe/Tyr ratio, showed any correlation with serum prolactin level. No association between Phe, Tyr, the Phe/Tyr ratio, and PRL was observed in female patients. The correlation coefficients are presented in Table 2. Figure 1a, b shows the relationships between Phe and PRL in the female and in the male subgroups.

**Table 2:** Correlation coefficients and *p* values between serum prolactin and Phe-Tyr levels in adult patients with PKU

	Female		Male	
	Correlation coefficient	*p* value	Correlation coefficient	*p* value
Phe	−0.082	0.478	0.177	0.230
Tyr	0.065	0.580	−0.231	0.114
Phe/Tyr	−0.134	0.251	0.252	0.084

Spearman’s rho correlation analysis. The significance level was set at 0.01

Dividing our patients groups into quartiles based on the Phe level, no significant differences in prolactin concentration were observed (Table 3). In the male group, the lowest Phe-quartile was not significantly different from the highest Phe-quartile (*p* = 0.366). In the female group, the lowest Phe-quartile was also not significantly different from the highest Phe-quartile (*p* = 0.532).

**Table 3:** Concentration of Phe and prolactin in patients divided into quartiles based on Phe concentration

	Female			Male		
	Lowest quartile	Highest quartile	*p* value	Lowest quartile	Highest quartile	*p* value
Phe (μmol/l)	246 (124–397)	1112 (1024–1121)	0.000	284 (183–404)	1068 (1021–1259)	0.001
Prolactin (ng/ml)	16 (7–37)	11 (8–31)	0.532	7 (3–14)	9 (5–47)	0.366

Data are given as median (minimum–maximum)

## Discussion

Prolactin is unique among the pituitary gland hormones because its secretion is mainly under negative tonic inhibition by hypothalamic dopamine [10]. Since dopamine biosynthesis begins from tyrosine, and its intracerebral availability depends on phenylalanine metabolism, it was hypothesized that prolactin secretion might be altered by the hyperphenylalaninemia observed in PKU.

To date, only three studies involving a relatively small study population evaluated the relationship between Phe and PRL in PKU. On the other hand, these studies were performed in children and adolescents. Schulpis et al. found a positive correlation between Phe and PRL serum levels [16], but Denecke et al. did not confirm these associations [6]. In the study by Schulpis et al., girls with high Phe levels complained of irregular menstruation, suggesting that other underlying diseases might be responsible for the increased PRL level [16]. Recently, van Vliet et al. examined the effect of BH4-treatment on prolactin secretion in a few BH4-responsive male PKU patients. Their findings revealed that prolactin concentration positively correlated with blood Phe level, and the BH4-treatment lowered the Phe and PRL levels [12].

Our data obtained in a larger number of adult PKU patients showed no correlation between Phe and prolactin serum concentration. In addition, neither phenylalanine, tyrosine nor the Phe/Tyr ratio correlated with the prolactin concentration.

To interpret our results, we should take into account more factors. First, the Phe level in the total and also in the gender-specific subgroups was around the upper limit established for adults (600 μmol/l). One might think that very high Phe levels (>1000 μmol/l, representing “loose diet” patients) can increase the prolactin level. Therefore, we divided our treated patients into different quartiles based on Phe concentration and compared the PRL concentration between these subgroups. Again, no significant differences in prolactin level was detected. It should be highlighted that the studied population had some dietary restrictions that lowered serum Phe concentration. An optimal comparison would be with a group of PKU patients absolutely free of diet control.

Second, PKU itself is not a homogeneous disease, as the mutation and the enzyme activity influence the severity of PKU. However, enzyme activity determination is not a realistic goal in all PKU patients, whereas comparing the hormone results according to the *PAH* gene mutation might reveal novel associations.

We also found that the tyrosine serum level did not correlate with the prolactin level. It is known that the serum Tyr level has relatively large diurnal fluctuations [17] mediated by diet and the phenylalanine-free protein substitutes used by patients. Therefore, the calculation with one sample concentration may not be enough to detect a possible correlation with prolactin. However, using blood samples drawn in standardized fasting conditions may eliminate the majority of these problems. Furthermore, the serum Tyr concentration cannot reflect the intracerebral Tyr concentration, which is the precursor of the intracerebral catecholamine synthesis. Hyperphenylalaninemia negatively influence dopamine utilization in the brain by competitive inhibition of the LNAA transporter and by reduced activity of DOPA decarboxylase [18]. Although there are data showing that the serum Phe level could correspondingly represent brain Phe concentration in PKU with great inter-individual variability [19, 20], no study performed in humans has been published that can give us an estimation of the suppressive effect of intracerebral Phe concentration on dopamine synthesis. Another limitation of our study may be that we did not take into account the menstrual cycle. However, the serum PRL level shows only minor fluctuations during the menstrual cycle, and therefore, this fluctuation may have a limited role in this current study. It should be mentioned that the strength of our study is the relatively high number of adult patients.

## Conclusion

Taken together, we found no correlation between serum Phe and prolactin in a relatively large, treated adult PKU population.

## Abbreviations

BBB: blood–brain barrier
BH4: tetrahydrobiopterin
LNAA: large neutral amino acid
PAH: phenylalanine hydroxylase
PEG: polyethylene glycol
Phe: phenylalanine
PKU: phenylketonuria
PRL: prolactin
TSH: thyroid-stimulating hormone
TRH: thyrotropin releasing hormone
Tyr: tyrosine
